# CO Inversion on a NaCl(100) Surface: A Multireference Quantum Embedding Study

**DOI:** 10.1021/acs.jpca.2c05844

**Published:** 2023-02-17

**Authors:** Nan He, Meng Huang, Francesco A. Evangelista

**Affiliations:** Department of Chemistry and Cherry Emerson Center for Scientific Computation, Emory University, Atlanta, Georgia 30322, United States

## Abstract

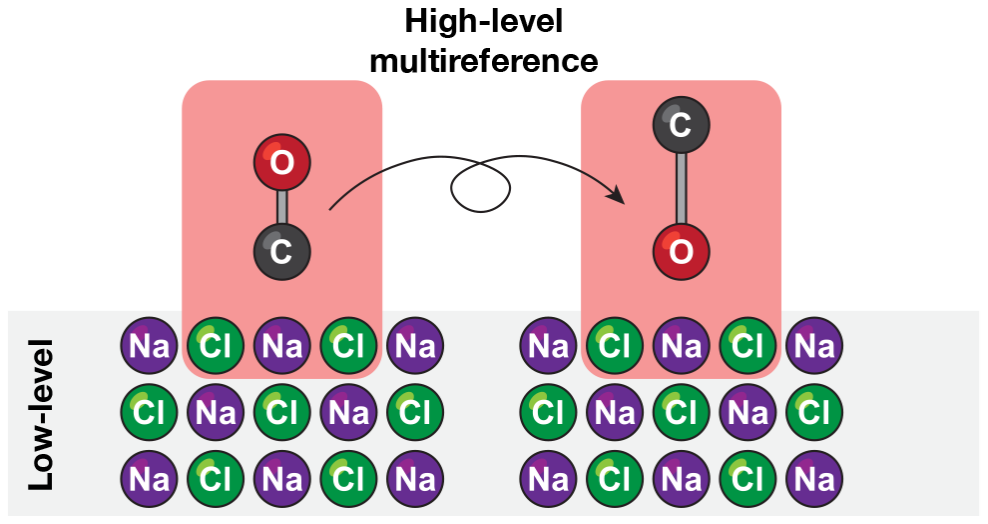

We develop a multireference quantum embedding model to investigate a recent experimental observation of the isomerization of vibrationally excited CO molecules on a NaCl(100) surface [*Science***2020**, *367*, 175–178]. To explore this mechanism, we built a reduced potential energy surface of CO interacting with NaCl(100) using a second-order multireference perturbation theory, modeling the adsorbate–surface interaction with our previously developed active space embedding theory (ASET). We considered an isolated CO molecule on NaCl(100) and a high-coverage CO monolayer (1/1), and for both we generated potential energy surfaces parametrized by the CO stretching, adsorption, and inversion coordinates. These surfaces are used to determine stationary points and adsorption energies and to perform a vibrational analysis of the states relevant to the inversion mechanism. We found that for near-equilibrium bond lengths, CO adsorbed in the C-down configuration is lower in energy than in the O-down configuration. Stretching of the C–O bond reverses the energetic order of these configurations, supporting the accepted isomerization mechanism. The vibrational constants obtained from these potential energy surfaces show a small (< 10 cm^–1^) blue- and red-shift for the C-down and O-down configurations, respectively, in agreement with experimental assignments and previous theoretical studies. Our vibrational analysis of the monolayer case suggests that the O-down configuration is energetically more stable than the C-down one beyond the 16th vibrational excited state of CO, a value slightly smaller than the one from quasi-classical trajectory simulations (22nd) and consistent with the experiment. Our analysis suggests that CO–CO interactions in the monolayer play an important role in stabilizing highly vibrationally excited states in the O-down configuration and reducing the barrier between the C-down and O-down geometries, therefore playing a crucial role in the inversion mechanism.

## 1 Introduction

Although molecular isomerization plays an important role in chemistry, direct quantum-state resolved observations of this process are elusive.^1^ Recently, the Wodtke group examined the isomerization of CO on a NaCl surface following infrared excitation and vibrational pooling.^2^ Their vibrational emission spectra showed signals for highly excited C-down and O-down isomers, indicating that isomerization proceeds via highly excited vibrational states with the driving force being attributed to CO dipole moment reversal when the C–O bond is elongated. A theoretical understanding of this isomerization process requires the construction of an accurate potential energy surface for the CO–NaCl(100) system, including those configurations in which the C–O bond is stretched and the wave function may exhibit strong multideterminantal character.

The adsorption of CO on NaCl(100) has been studied extensively both experimentally and theoretically.^3−8^ Several recent theoretical studies have focused on simulating and interpreting the experimental observations of Lau et al.^2^ Chen, Hariharan, Meyer, and Guo^9^ computed potential energy surfaces (PESs) of CO on NaCl(100) under different CO coverage levels using a periodic model and density functional theory (DFT). This work showed that as CO is vibrationally excited the energy of the two isomers changes, and for highly excited vibrational states (ν ≈ 30) the O-down isomer becomes more stable than the C-down one. Sinha and Saalfrank^10^ confirmed this observation and computed vibrational eigenstates of two- and three-dimensional models based on a periodic DFT potential they constructed. Nandi et al.^11^ used a finite CO–NaCl cluster model to study the dynamics of CO isomerization with quasi-classical trajectories. Their work suggested that isomerization may happen at larger CO–NaCl distances than the isomerization saddle point, consistent with a roaming mechanism.^12−14^

The computational modeling of molecules interacting with surfaces presents several challenges. One is describing isolated local geometry changes (dissociation, desorption), which may become costly with periodic boundary computations due to the need of large unit cells. Quantum embedding theories solve this problem by partitioning the system into a fragment and environment. These two portions of the system can then be treated at different levels of theory, reducing the overall computational cost.

Various quantum embedding schemes have been developed^15−25^ and successfully applied to large chemical systems,^26−33^ including molecules interacting with surfaces.^34−38^ If the electronic state of the fragment develops multideterminantal character (like in bond-breaking processes or excited states), it might be necessary to treat the fragment with a high-level multireference method in combination with a multireference quantum embedding scheme. A multireference treatment is advantageous because it can predict quantitatively accurate potential energy surfaces far from the equilibrium geometry for both ground and excited states, even in the presence of near-degeneracies, avoiding the breakdown displayed by single-reference electronic structure methods.

In this work, we revisit the CO inversion problem from the point of view of multireference electronic structure theory. We combine a multireference description of a CO-NaCl cluster based on the second-order driven similarity renormalization group (DSRG-MRPT2)^39,40^ with our recently introduced active space embedding theory (ASET)^41^ and augment this treatment with a classical potential that accounts for electrostatic and dispersion interactions with the bulk. This combination of theory offers a good compromise between cost of the computation and the accuracy of the potential. The multireference treatment employed in this work is particularly useful to compute an accurate potential energy curve of CO interacting with NaCl over a broad range of bond distances, especially in the high-energy region relevant to the inversion mechanism of vibrationally excited CO. Other aspects investigated in this work, including the structure and adsorption energy of CO, can be studied even with single-reference wave function and DFT methods.^7,9,11^

This article is organized as follows: In Section 2, we briefly review the ASET embedding scheme and discuss the details of our quantum-mechanical computations and vibrational analysis. Section 3 reports the details of the ASET computations on the CO-NaCl(100) system. In Section 4.1, we compare different embedding models of CO on NaCl(100) for an isolated molecule and a high surface coverage case (monolayer). These models are then used in Sections 4.2–4.5 to examine the optimized geometry, characterize the PES, and analyze the vibrationally excited states of these two systems. In Section 5, we summarize the results and discuss the insights gained from this study.

## 2 Theory

### 2.1 Embedding Model for CO Absorbed on NaCl

This section describes the multilevel embedding model used to compute the PES of CO adsorbed on a NaCl surface. Because modeling the CO molecule over a broad range of bond lengths is key to understanding the isomerization process, we start from a complete-active-space self-consistent field (CASSCF) calculation of CO and a small NaCl cluster. Following Boese and Saalfrank,^7^ we consider a cluster containing two 3 × 3 NaCl layers, with a Na central atom in the surface layer closest to the CO. In Table S1 of the Supporting Information we provide a comparison of the structure, vibrational frequency, and adsorption energy of CO for various clusters. These data show that two 3 × 3 layers are sufficient to recover a converged structure and vibrational frequency but are insufficient to compute the adsorption energy. As described below, the remaining interactions with the surface are treated with a classical potential.

**Figure 1:**
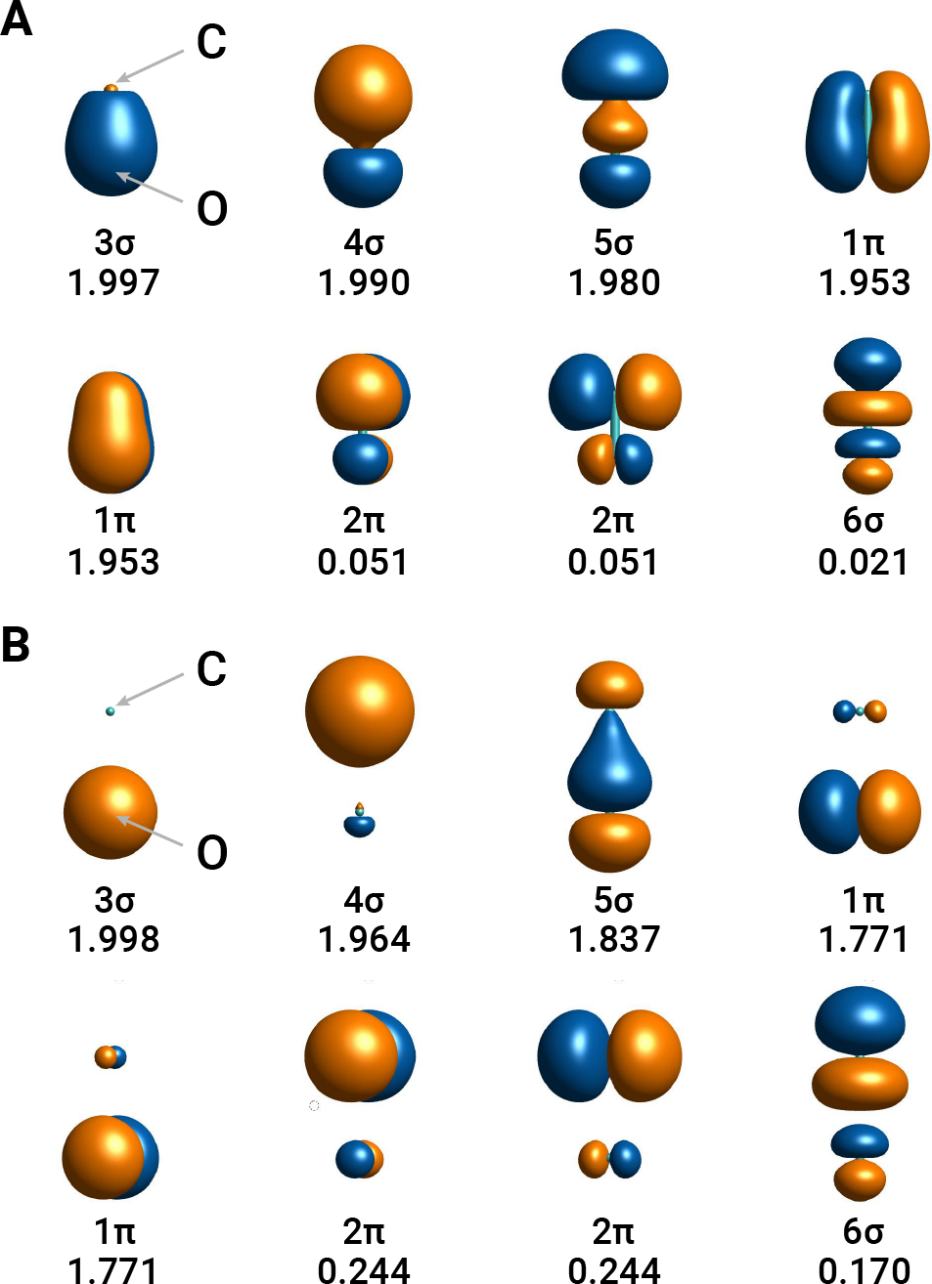
Natural active orbitals of CO around the equilibrium bond distance (A, *r* = 1.15 Å) and in the recoupling region (B, *r* = 1.95 Å). The active orbitals are computed at the CASSCF(10e,8o)/cc-pCVTZ level of theory starting from orbitals selected via the AVAS procedure (see ref (42)). For each orbital we report the corresponding symmetry label and natural occupation number.

We choose an active space that includes eight valence orbitals of CO that maximize the overlap with the atomic 2s and 2p orbitals of C and O, using the atomic valence active space (AVAS) method.^42^ When applied to an isolated CO molecule, this procedure yields the natural active orbitals shown in Figure 1. In our quantum embedding computations, these orbitals are always included in the active space of our high-level multireference treatment.

After generating CASSCF orbitals, we apply the mean-field version of ASET [ASET(mf)].^41^ The ASET(mf) scheme is used to localize and separate orbitals into two sets: fragment (A) and environment (B) orbitals.^41,43^ This step requires the user to specify a list of atoms assigned to the fragment. To accurately reproduce the interaction of CO with the surface, the fragment space should contain the orbitals of CO and the closest Na and Cl atoms. After partitioning and localization, ASET produces an effective Hamiltonian for the fragment, which provides the input Hamiltonian in the form of integrals to a high-level multireference theory. The mean-field version of ASET accounts for the fragment–environment interaction with a static effective one-electron potential. Although this treatment neglects instantaneous fragment–environment fluctuations, a study that introduced this missing effect at the second-order level in perturbation theory found only a small improvement in the energetics.^43^ The present embedding method is well suited for insulator surfaces. To enable the treatment of molecules adsorbed on metal surfaces, an extension of the embedding scheme is necessary to correctly treat periodic boundary conditions and screening effects, for example, via the random phase approximation. Several researches have already explored embedding approaches along these directions.^44,45^

Our high-level multireference treatment is based on the unrelaxed version of the DSRG-MRPT2.^39,40^ One feature that distinguishes the DSRG is a controllable diagonalization of the Hamiltonian that helps avoid potential energy surface discontinuities caused by intruder states. The extent to which dynamical correlation is accounted for by the DSRG-MRPT2 is controlled by the so-called flow parameter *s*,^46^ which in this work we set to the default recommended value (0.5 *E*_h_^–2^). Compared to other multireference perturbation theories like CASPT2 and NEVPT2,^47,48^ DSRG-MRPT2 has the advantage of being robust to small denominators and requiring fewer computational resources, as the energy depends only on the three-body density matrix of the reference.^39,47,48^ Benchmarks show that these advantages come at the expense of slight loss of accuracy compared to CASPT2 and NEVPT2.^39,49,50^

**Figure 2:**
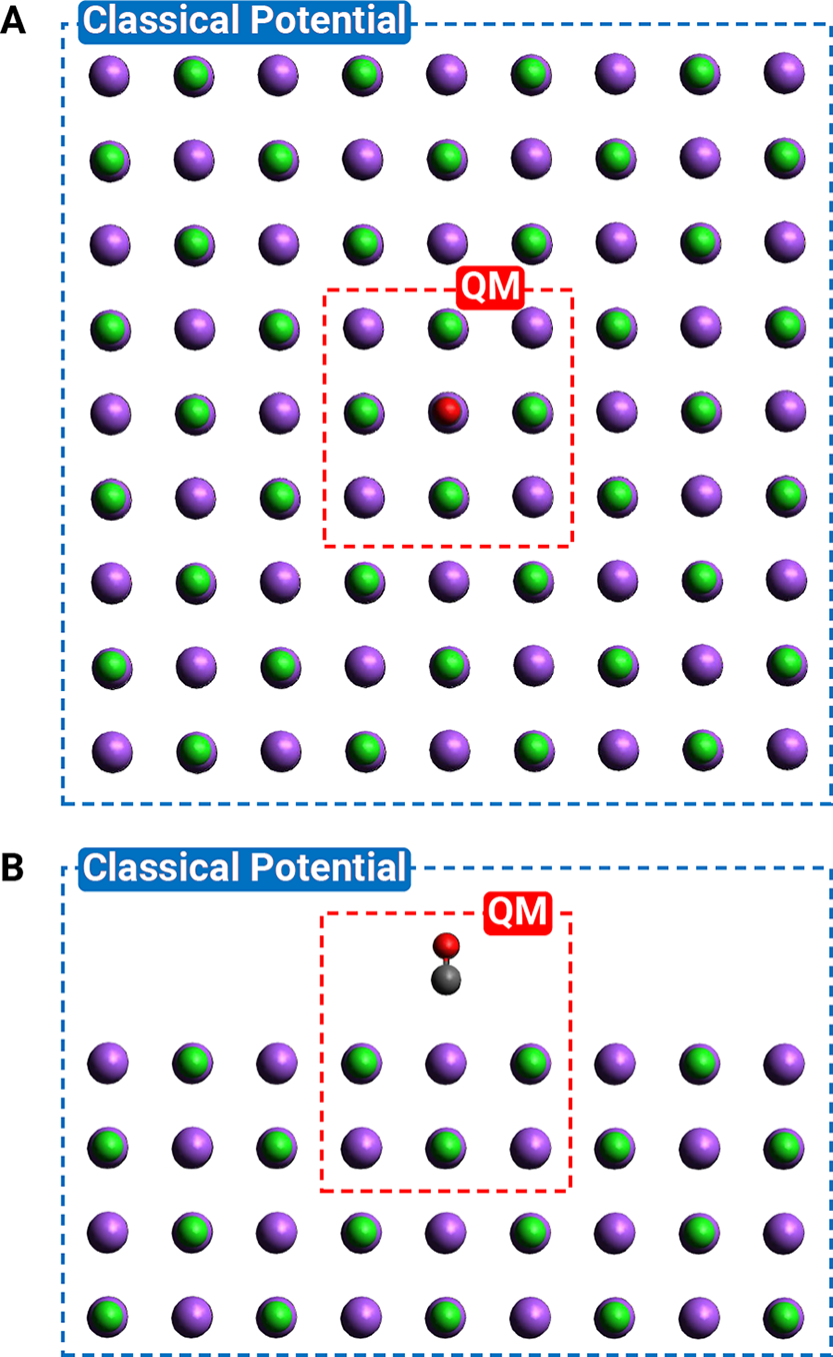
Top (A) and side (B) view of the model for CO-NaCl(100) used in this work (showing only the first layer). The quantum mechanical region (QM) is treated with a high-level multireference method and the ASET(mf) embedding scheme. The surrounding classical potential region accounts for electrostatic and dispersion interactions. Carbon and oxygen atoms are represented with gray and red, while Na^+^ and Cl^–^ ions are colored with purple and green.

Lastly, to simulate the long-range interactions between CO and NaCl both along the surface and deep into the bulk, we include the electrostatic (external) potential due to surrounding Na^+^ and Cl^–^ ions treated as point charges plus corrections for dispersion interactions. As shown in Figure 2, this classical external potential accounts for replicas of the finite NaCl cluster included in the quantum mechanical computation (four layers containing 7 × 7 Na_9_Cl_9_ clusters, for a total of 1755 Na^+^ and 1755 Cl^–^ atoms). The details of how the external potential is constructed and the errors associated with truncating the NaCl slab are discussed in the Supporting Information. To model a CO monolayer, we use a recently developed potential energy surface for the CO dimer, obtained from all-electron CCSD(T)-F12b computations with complete basis set limit extrapolation (see details in Section 4.3).^51^

### 2.2 Vibration Models

**Figure 3:**
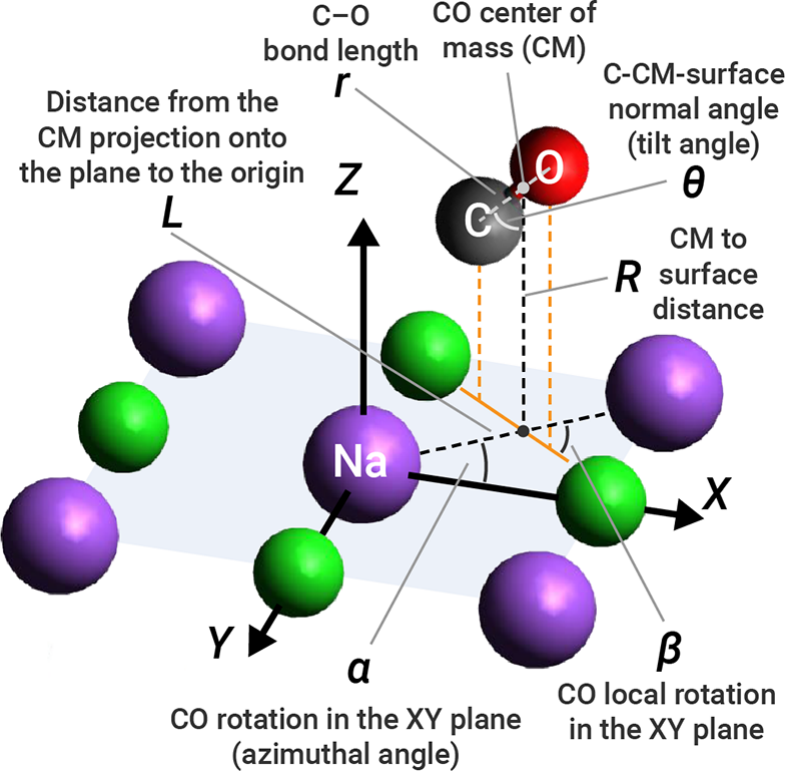
Definition of the six coordinates used in this work and that encompass all arrangements of CO on a NaCl(100) surface.

In order to investigate the inversion of highly vibrationally excited CO, we computed the vibrational eigenstates using three reduced-dimensionality models. Keeping the position of the Na and Cl atoms fixed, there are six degrees of freedom for the C and O atoms; in this paper, we decompose the coordinates of CO as a center-of-mass (CM) plus rotations and C–O bond stretching. The six coordinates we employ are shown in Figure 3.

The simplest vibrational model accounts only for the CO stretching mode along the C–O distance (*r*). The corresponding vibrational Hamiltonian is simply
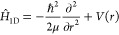
1where μ is the reduced mass of CO and *V*(*r*) is the potential computed keeping the other five coordinates fixed.

The other two vibrational models account for the couplings of the CO stretching mode with other degrees of freedom and are taken from a previous study^10^ on CO–NaCl based on a DFT potential. The first one involves the C–O distance and the CO tilt angle (θ, also termed inversion coordinate), where θ = 0° corresponds to the C atom pointing to the surface. The corresponding model Hamiltonian, *Ĥ*_2D(*r*,θ)_ is
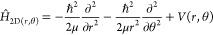
2Here the kinetic energy coupling between the two vibrational coordinates is represented by the dependence on *r* of the moment of inertia (μ*r*^2^). The second 2D vibrational model involves the CO stretching mode and *Z*-axis translation of the CO center of mass (*R*). The corresponding model Hamiltonian, *Ĥ*_2D_(*r*,*R*) can be written as
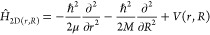
3where *M* is the total mass of the CO molecule. These 2D models ignore other degrees of freedom such as CO rotation along the azimuthal angle (α), which can potentially limit the accuracy of our description of the CO isomerization process and fail to capture minima with a spiral structure, as observed by Boese and Saalfrank.^7^

To find the eigenstates of these three model Hamiltonians, we employ the one- and two-dimensional Discrete Variable Representation (DVR) method, using the representation of Colbert and Miller.^52^ We impose periodic boundary conditions on the tilt angle (θ ∈ [−180°, 180°]) and use a grid of evenly spaced points. The number of grid points is 201 for the 1D model, 59 × 101 for the 2D(*r*,θ) model, and 59 × 59 for the 2D(*r*,*R*) model. The values of potential energy at each DVR grid point are evaluated using cubic spline interpolations of *ab initio* energies. The grids used provide a compromise between the ability to fully diagonalize the Hamiltonian matrix and the accuracy of the energy levels, which are converged to < 1 cm^–1^ for highly vibrationally excited states of CO up to quantum number 25, as shown in the convergence plot (Figure S2) reported in the Supporting Information.

## 3 Computational Details

All ASET and DSRG-MRPT2 computations reported in this work were performed with FORTE,^53^ using an efficient implementation based on density-fitted integrals.^49^ All computations used integrals, reference orbitals, and electrostatic potentials obtained from PSI4.^54^ For the CASSCF and DSRG-MRPT2 computations, the density fitting basis sets for C and O atoms are cc-pCVTZ-JKFIT (CASSCF) and cc-pCVTZ-RI (DSRG-MRPT2), while for Na and Cl atoms the smaller cc-pVDZ-JKFIT and cc-pVDZ-RI bases were selected.^55−57^ For the geometry comparison in Section 4.2, we use the def2-SVP basis.

To evaluate properties of CO-NaCl, we fit single-point energies to a polynomial in the variables *R* and cos(θ) such that the total degree of the polynomial is less than or equal to 12. The CO-NaCl PES is fitted using Scikit-learn’s polynomial regression module with cross-validation (0.8:0.2 split, 5 folds) to prevent overfitting. We ensure through cross-validation that all *R*^2^ scores are higher than 0.996, and the root-mean-square errors (RMSE) for all folds are smaller than 5 cm^–1^. The minima and transition states are found in this PES using the limited-memory Broyden–Fletcher–Goldfarb–Shanno algorithm (L-BFGS) in SciPy, with boundaries enforced using an exponential penalty beyond the *R* and *r* data range.^58,59^ The details of the fitting procedure can be found in the Supporting Information.

## 4 Results and Discussion

### 4.1 Benchmarking of the Computational Model

**Figure 4:**
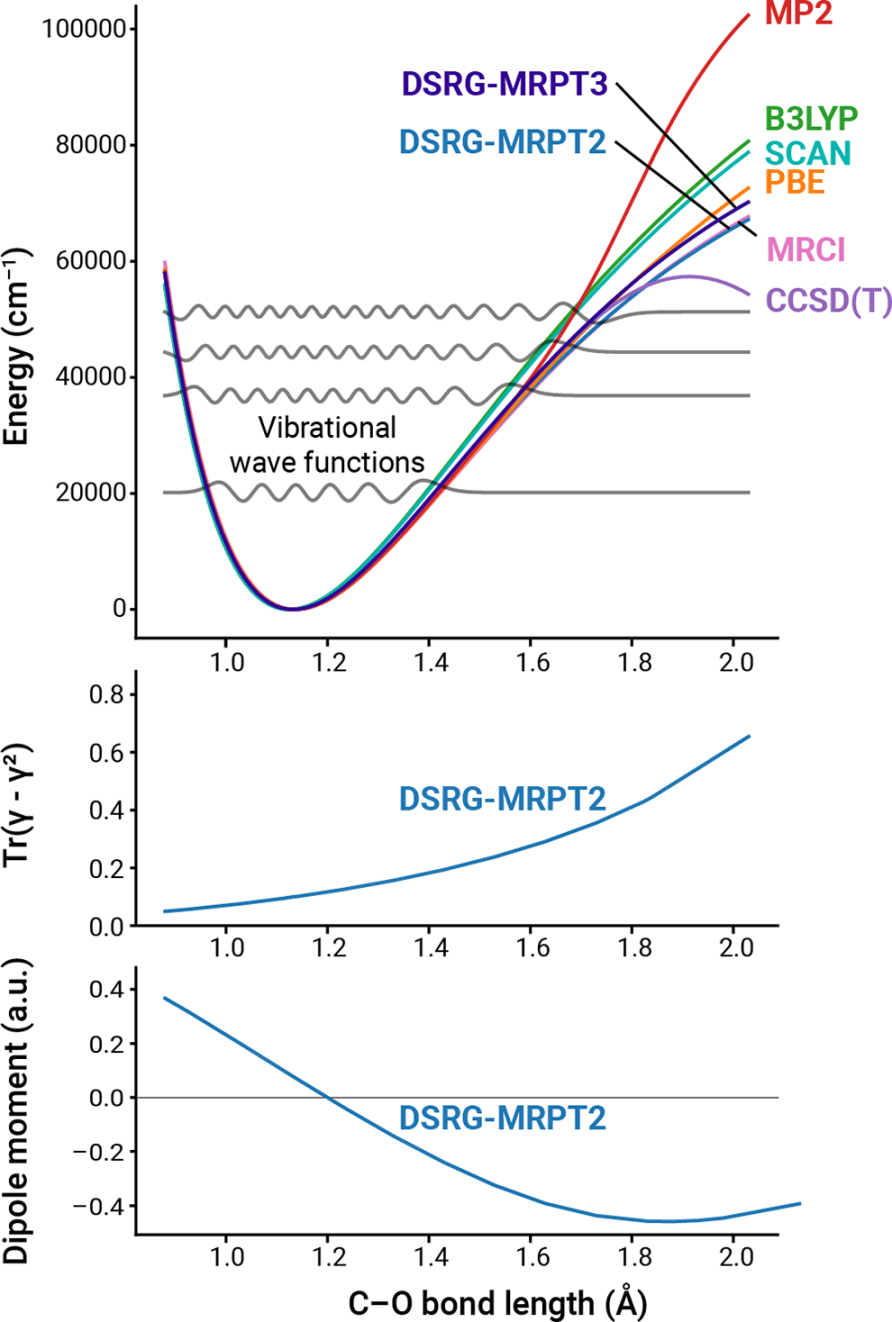
(top) Dissociation curve of a single CO molecule in the gas phase computed using various methods and the cc-pCVTZ basis set (except for the MRCI computations, which use the cc-pVTZ basis set). The curves are aligned with their respective minimum energy. The scan is done from 0.83 to 2.03 Å, using 50 points in total (using more points around 1.13 Å to capture features near the minimum). The vibrational wave functions (ν = 10, 20, 25, 30) computed using DSRG-MRPT2 and DVR are shown shown as gray lines. (middle) Deviation from idempotency of the one-electron CASSCF(10e,8o) density matrix (**γ**), defined as Tr(**γ** – **γ**^2^), a metric of the multideterminantal character of the reference state used in the DSRG-MRPT2 computations. (bottom) Dipole moment of CO computed with DSRG-MRPT2. Positive values correspond to the charge distribution C^δ−^–O^δ+^.

We begin by first benchmarking the accuracy of the ground-state potential energy curve of gas-phase CO computed with the DSRG-MRPT2 and comparing it to other electronic structure methods used in previous studies. In Figure 4, we show the potential curve of CO for bond distances ranging from 0.83 to 2.03 Å computed with three DFT functionals (PBE, B3LYP, SCAN),^60−63^ restricted second-order Møller–Plesset perturbation theory (MP2), restricted coupled cluster with singles, doubles, and perturbative triples [CCSD(T)], internally contracted multireference configuration interaction (MRCI), DSRG-MRPT2, and third-order DSRG (DSRG-MRPT3).^40^Figure 4 shows no major differences among these methods around the equilibrium distance; however, significant deviations start to appear in the recoupling region (*r* > ca. 1.5 Å). In particular, MP2 and CCSD(T) show major deviations after 1.62 and 1.75 Å, respectively. These deviations correlate well with a simple metric of multideterminantal character of the CASSCF state, the deviation from idempotency of the one-electron density matrix, which is plotted in the middle panel of Figure 4. At the equilibrium geometry, the deviation from idempotency is ca. 0.2, and this value increases rapidly in the recoupling region, tripling when *r* > 1.9 Å, in the region where MP2 and CCSD(T) deviate significantly from MRCI and DSRG-MRPT2. In the bottom panel of Figure 4, we report the dipole moment as a function of *r* for isolated CO computed with the DSRG-MRPT2 method. This plot shows that the CO goes from being polarized as C^δ−^–O^δ+^ to C^δ+^–O^δ−^ as *r* increases, with the switch between these two forms happening around 1.2 Å.

From the potential energy curves, we obtain vibrational constants from DVR computations (fitted to the first 25 vibrational levels). The results are shown in Table 1. Compared to the experimental value, MP2, CCSD(T), and MRCI generally underestimate ω_e_ by ca. 8 to 34 cm^–1^. The DSRG-MRPT2 results show a comparable deviation from the experimental frequency (ca. 23 cm^–1^). Increasing the correlation treatment in the DSRG to third order significantly improves the agreement with the experimental ω_e_, bringing the deviation down to only 3.2 cm^–1^. Although preferable to the DSRG-MRPT2, this higher level treatment is more expensive and is not further considered for the evaluation of the PES.

**Table 1:** Vibrational Constants of Gas-Phase ^12^C^16^O Computed Using Different Methods and the cc-pCVTZ Basis Set (Except for the MRCI Computations, Which Use the cc-pVTZ Basis Set)^a^

method	ω_e_ (cm^–1^)	ω_e_*x*_e_ (cm^–1^)	ω_e_*y*_e_ (10^–3^ cm^–1^)
PBE	2128.0	12.8	30.0
B3LYP	2212.8	12.5	26.9
SCAN	2206.4	12.7	25.3
MP2	2149.3	16.1	179.8
CCSD(T)	2161.6	12.8	2.1
DSRG-MRPT2	2147.2	13.5	8.4
DSRG-MRPT3	2166.6	13.1	10.6
MRCI^b^	2135.6	13.3	17.0
Exp^c^	2169.8	13.3	10.5

a Results are computed using a one-dimensional DVR and fits to the first 25 vibrational levels.

b MRCI potential taken from ref (11).

c Experimental values taken from ref (64).

**Figure 5:**
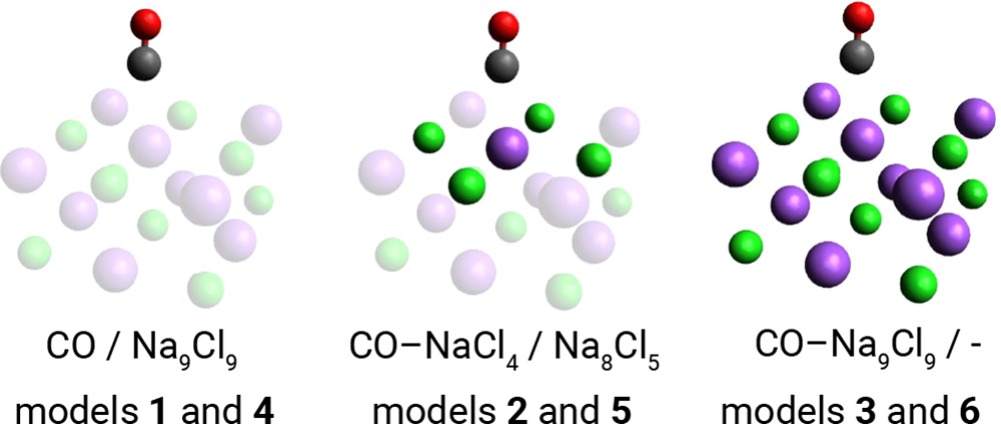
Different fragment–environment partitions tested in this work. Purple and green balls represent Na^+^ and Cl^–^ ions, respectively. Semitransparent atoms are included in the ASET environment.

In the next step, we identify a model for the CO–NaCl(100) system that is sufficiently accurate and computationally feasible. In Figure 5, we show the two fragment/environment partitions of a Na_9_Cl_9_ cluster used in the embedding approach and the full Na_9_Cl_9_ cluster. From these, we generate six models and compare their properties with previous experimental and DFT results.^2,9^ Models **1** and **2** are based on ASET and include in the definition of the fragment CO and CO–NaCl_4_, respectively. Model **3** contains the entire Na_9_Cl_9_ cluster, and it is equivalent to a full correlated computation without embedding. Models **4**–**6** correspond to models **1**–**3** augmented with a classical external potential that includes electrostatic plus dispersion corrections, previously mentioned in Section 2.

In Table 2, we report the definition of the six models and corresponding properties of the CO molecule together with previous DFT results and experimental data for the monolayer case.^2,9^ For each model, we compute the equilibrium center-of-mass distance from the surface for C-down adsorption [*R*(C-d)] and O-down adsorption [*R*(O-d)], the fundamental CO stretching frequency for C-down [ν_0_(C-d)] and O-down adsorbed [ν_0_(O-d)] CO, and the difference between the two fundamental CO stretching frequencies, Δν_0_ = ν_0_(C-d) – ν_0_(O-d). The equilibrium *R* value is determined by a potential energy scan with 0.01 Å spacing and *r* fixed at 1.132 Å assuming a perpendicular CO geometry aligned with the central Na^+^ (corresponding to *L* = 0). The corresponding fundamental CO stretching frequency is computed via a 1-dimensional DVR based on a 15-point potential computed in the range *r*_e_ – 0.3 Å to *r*_e_ + 0.4 Å at the equilibrium *R* value.

**Table 2:** Comparison of the Embedding Models Shown in Figure 5 for ^12^C^16^O^a^

model	fragment	environment	CP	*R*(C-d)	*R*(O-d)	AE	ν_0_(C-d)	ν_0_(O-d)	Δν_0_
**1**	CO	Na_9_Cl_9_	no	3.65	3.53	389	2127.6	2118.2	9.4
**2**	CO–NaCl_4_	Na_8_Cl_5_	no	3.41	3.15	1320	2130.8	2118.7	12.1
**3**	CO–Na_9_Cl_9_		no	3.32	3.07	1683	2134.0	2119.7	14.3
**4**	CO	Na_9_Cl_9_	yes	3.44	3.16	1063	2135.7	2118.2	17.5
**5**	CO–NaCl_4_	Na_8_Cl_5_	yes	3.38	3.12	1576	2135.9	2116.6	19.3
**6**	CO–Na_9_Cl_9_		yes	3.31	3.05	1898	2138.2	2117.5	20.7
DFT^b^				3.33	3.11	1502	2136.1	2121.6	14.5
Exp^c^						1504	2150.2		

a Bond distances are in units of Å while the CO adsorption energy (AE) and vibrational frequencies are in cm^–1^. The value of *R* is optimized using a one-dimensional scan where all other coordinates are fixed. CP stands for classical potential. The ν_0_(C-d) and ν_0_(O-d) are computed using a 1-D DVR along *r* using 100 points between 0.83 and 1.83 Å. The potential used in the DVR computation is obtained by cubic spline interpolation of 21 equidistant points.

b Data taken from ref (9), converting the 1/8 PBE results to ^12^C^16^O via the reduced mass.

c Data for a CO monolayer taken from ref (2).

As can be seen from Table 2, embedding models that include only the CO in the high-level region (**1** and **4**) fail to accurately predict the equilibrium *R* value and adsorption energy, indicating that an explicit treatment of the quantum mechanical interaction of CO with the surface atoms is necessary to recover these features. The ASET-based models **2** and **5** treat only the fragment [CO–NaCl_4_]^3–^ at the DSRG-MRPT2 level and accurately reproduce the structure and fundamental frequencies from DFT. The inclusion of the classical potential in model **5** further improves the adsorption energy (from 1320 to 1576 cm^–1^), bringing it close to the DFT and experimental values (1502 and 1504 cm^–1^, respectively). The properties obtained from the most elaborate models (**3** and **6**) are in excellent agreement with previous DFT results, with *R*(C-d) differing only by 0.02 Å. However, models **3** and **6** tend to overestimate the adsorption energy by several hundred cm^–1^. When comparing the cost of models **5** and **6**, we find that computations with the latter are around 7–8 times more expensive than the former due to the larger number of correlated MOs (176 vs 410, respectively). Therefore, due to its good compromise between accuracy and cost, we will employ model **5** for our full PES computations on an isolated CO molecule on NaCl(100).

### 4.2 Optimized Geometry of a Single CO Adsorbed on NaCl(100) Surface

Using the embedding model **5**, we optimize the geometry of both C-down and O-down configurations with a grid-search strategy combined with polynomial interpolation and found that the global minimum in both cases is a perpendicular geometry with *r* = 1.1323 Å, *R* = 3.3797 Å for C-down (θ = 0°) and *r* = 1.1340 Å, *R* = 3.1242 Å for O-down (θ = 180°), with *L* = 0 (and, therefore, α and β arbitrary) in both cases. This result is in agreement with a previous theoretical study by Meredith and Stone,^4^ which predicted that an isolated CO molecule adopts a perpendicular geometry with the C atom above a Na^+^ ion, and the more recent work of Boese and Saalfrank.^7^ In contrast, for a monolayer both experiments^3^ and theory^4,6,9,11^ suggest that at low temperatures (below 35 K) the CO molecules are tilted with two possible minima (1 × 1 and 2 × 1). Furthermore, above 35 K, the CO molecules adopt a 1 × 1 structure with all molecules perpendicularly aligned to the surface. This structure is estimated by theory to lay only 32 cm^–1^ above the most stable tilted monolayer structure.

**Figure 6:**
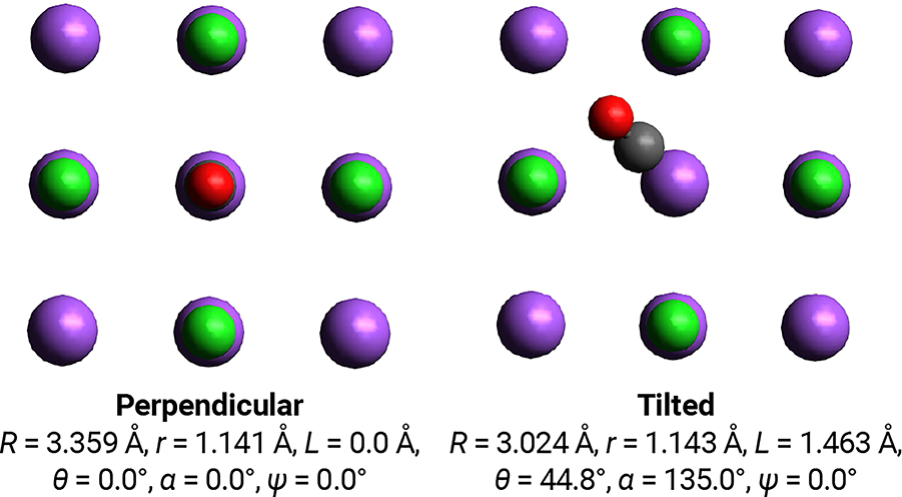
Perpendicular and tilted geometries optimized geometries of the CO–Na_9_Cl_9_ cluster computed at the PBE/def2-SVP. The coordinates used are defined in Figure 3.

We have found that it is rather difficult for *ab initio* theories to accurately capture the energy difference between the perpendicular and tilted geometry of a single CO molecule on a NaCl(100) surface. To illustrate this point, we consider two stationary points on the PBE/def2-SVP potential energy surface for the CO–Na_9_Cl_9_ cluster: the perpendicular and tilted geometries shown in Figure 6 (obtained by keeping the Na and Cl atom positions frozen). For both geometries we then computed the energy difference between the two geometries, Δ*E* = *E*_tilted_ – *E*_perpendicular_, using various DFT functionals (PBE, SCAN, B3LYP, and PBE0)^60−63,65^ optionally including Grimme’s D3 dispersion corrections,^66^ MP2, and ASET(mf)-[DSRG-MRPT2/3] based on model **5**,^39−41^ and full DSRG-MRPT2 computations. As shown in Table 3, there is a wide spread in the energy difference. The PBE, B3LYP, and SCAN functionals predict that the tilted geometry is significantly lower in energy, while PBE0 only slightly favors the tilted one. Adding the empirical dispersion correction (D3) leads to a prediction that the tilted geometry is more stable for all functionals; however, the magnitude of the D3 correction ranges from a slight destabilization (by 5 cm^–1^ for SCAN) to stabilization up to 288 cm^–1^ (B3LYP) of the tilted geometry. In contrast, MP2 and the ASET-DSRG models agree with the theoretical prediction of a more stable perpendicular geometry for isolated CO.^4−7^ Because all the results from wave function methods do not account for dispersion interactions with Na^+^ and Cl^–^ ions outside the 3 × 3 cell, we have also analyzed the long-range dispersion interaction in the external potential and found a 20.8 cm^–1^ stabilization of the tilted geometry for the first NaCl layer and a correction of < 0.4 cm^–1^ due to the second and further layers.

**Table 3:** Energy Difference between the C-Down Perpendicular and Tilted Geometries (*E*_tilted_ – *E*_perpendicular_) Computed Using Different Methods^a^

method	*E*_tilted_ – *E*_perpendicular_ (cm^–1^)
PBE	–174.8
PBE-D3	–291.6
SCAN	–618.6
SCAN-D3	–613.5
B3LYP	–258.2
B3LYP-D3	–545.8
PBE0	–17.2
PBE0-D3	–172.0
MP2	233.2
ASET(mf)-[DSRG-MRPT2]	329.5
ASET(mf)-[DSRG-MRPT3]	176.3
full DSRG-MRPT2	329.1

a All results include a classical electrostatic potential consisting of one layer (153 Na^+^ and 153 Cl^–^ ions) but no additional dispersion energy corrections.

### 4.3 Potential Energy Surface for CO Inversion

**Figure 7:**
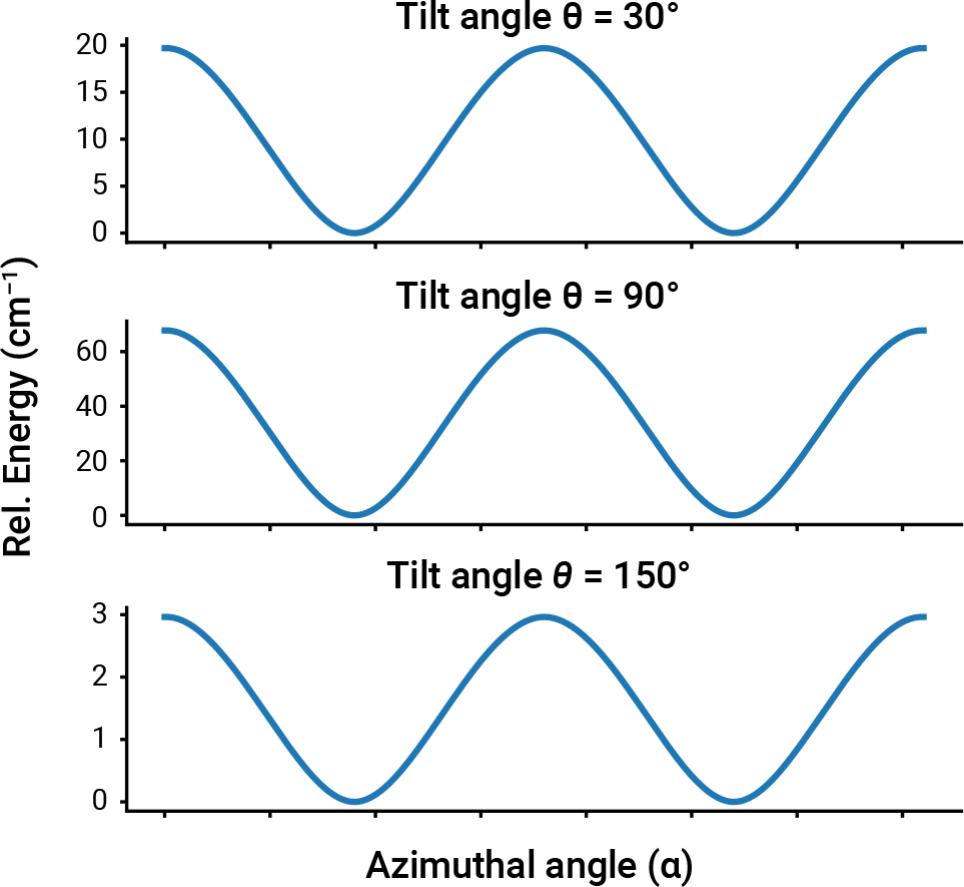
Isolated CO–NaCl(100) potential energy as a function of the azimuthal angle computed using model **5** at the ASET(mf)-[DSRG-MRPT2]/cc-pCVTZ level of theory.

In this section, we investigate the features of the PES of CO adsorbed on NaCl(100). We begin by discussing the inversion of an isolated CO and then extend our analysis to a CO monolayer. The starting point of our investigation on the isolated CO is the perpendicular minimum geometry of model **5**. We first investigate the dependence of potential energy as a function of the azimuthal angle (α) for three values of the tilt angle (θ = 30°, 90°, 150°). During the potential energy scan, we keep the projection of the CO center-of-mass on the surface fixed onto the central Na^+^ ion by imposing *L* = 0 and maintain the CO center-of-mass distance from the surface fixed at the optimum distance for the C-down configuration. Figure 7 shows the results of this analysis. We generally note that for all three tilted configurations there are four equivalent minima corresponding to diagonal orientations of the CO molecule (α = 45° + *k*90°, with *k* = 0, 1, 2, 3). The energy profiles at different tilt angles are qualitatively similar. However, the magnitude of the energy barrier ranges from as low as ca. 3 cm^–1^ (for θ = 150°) to ca. 67 cm^–1^ (for θ = 90°).

On the basis of these considerations, we build a three-dimensional potential energy surface involving the C–O bond length (*r*), the distance of the CO center of mass from the NaCl(100) surface (*R*), and the tilt angle (θ) defined as the angle between the C–O vector and the NaCl(100) normal vector. During the potential energy scan, we fix α + β = 45° and *L* = 0, which corresponds to a CO oriented diagonally with respect to the unit cell. Our potential energy surface covers *r* from 0.832 to 1.832 Å, *R* from 2.83 to 4.93 Å, and θ from 0° to 180°. All data are computed at the ASET(mf)-[DSRG-MRPT2] level of theory, with empirical dispersion and external charge fields included. A total of 5376 points are computed for the PES, and the scan takes < 50 h using 288 CPU cores.

**Figure 8:**
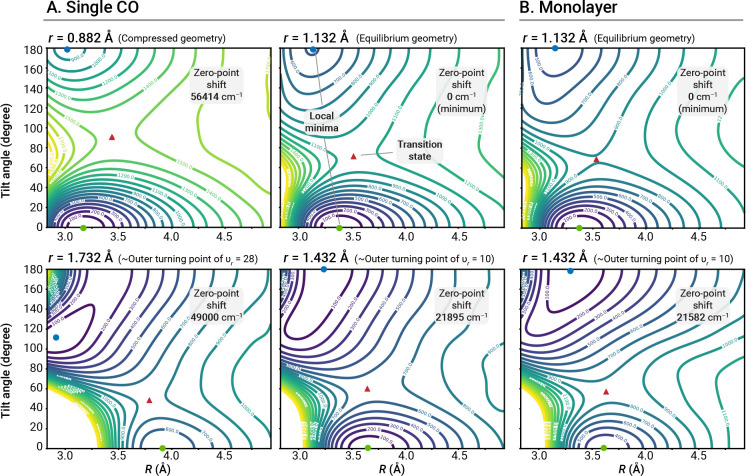
Potential energy surfaces cross sections for (A) the isolated CO model and (B) a ^12^C^16^O monolayer as a function of the CO tilt angle (θ) and CO center of mass-surface distance (*R*, in Å) computed using ASET(mf)-[DSRG-MRPT2]/cc-pCVTZ for different values of the C–O distance (*r*, in Å). Contour lines are separated by 100 cm^–1^. The blue and green circles mark the O-down and C-down minima, while the red triangles mark transition states. The energy shifts with respect to the C-down isomer for each cross section are indicated in each quadrant.

Figure 8A shows four slices of the PES at C–O bond lengths *r* = 0.882, 1.132, 1.432, and 1.732 Å using contour lines at 100 cm^–1^ energy intervals. These values of the C–O bond length correspond to a very compressed bond (0.882 Å), the equilibrium geometry (1.132 Å), and the outer classical turning points of gas-phase CO with stretching mode quantum number (ν_*r*_) approximately equal to 10 (1.432 Å) and 28 (1.732 Å). Both C-down (θ = 0°) and O-down (θ = 180°) CO geometries correspond to minima, with the former being the global one at *r* = 1.132 Å and *R* = 3.42 Å. There are several important observations that can be made about the PES. First, the value of *R* on the minimum-energy path of CO stretching motion varies significantly. At *r* = 0.882 Å, the C-down and O-down configurations display minima at *R* = 3.18 and 3.02 Å, respectively. When the *r* is increased to 1.132, 1.432, and 1.732 Å, the optimal value of *R* for C-down CO increase to 3.38, 3.65, and 3.92 Å, respectively. In contrast, the optimal *R* value for O-down CO does not display such large variations, and its stable adsorption basin becomes flatter with larger *r* values.

A second important observation concerns the energy difference between the C-down and O-down equilibrium geometries, Δ*E*_iso_ = *E*(O-down) – *E*(C-down), which is relevant to the isomerization mechanism. Our PES predicts this energy difference to be 490 cm^–1^ (without zero-point vibrational corrections and at optimal *r* and *R* values). This value is smaller than the one predicted by Nandi and co-workers^11^ (729 cm^–1^) and an electrostatic model (750 cm^–1^);^67^ however, it is in good agreement with the PBE (561 cm^–1^) and PBE-D3 (614 cm^–1^) results of Chen and co-workers^9^ as well as the PBE-D2 (624 cm^–1^) result of Sinha and Saalfrank.^10^ At *r* = 0.882 Å, the C-down geometry is energetically more stable and Δ*E*_iso_ = 867 cm^–1^. However, at *r* = 1.432 Å, the O-down geometry becomes slightly more stable than the C-down, with Δ*E*_iso_ = −6.8 cm^–1^. When *r* = 1.732 Å, the O-down geometry is 502 cm^–1^ lower in energy than the C-down one, and the O-down minimum becomes tilted. This stabilization was observed in previous studies,^9^ where PBE computations on periodic models [both p(1 × 1) and p(2 × 1)] predict the O-down geometry to be the most stable past *r* = 1.59 Å.^9^

Finally, the height of the barrier for the C- to O-down isomerization along the θ coordinate decreases as *r* increases. When *r* = 0.882 Å, the inversion barrier is 1495 cm^–1^, and it decreases to 1075, 630, and 276 cm^–1^ when *r* = 1.132, 1.432, and 1.732 Å, respectively. Therefore, the inversion between C-down and O-down geometries is facilitated by a lowering of the barrier when the C–O bond length is stretched to 1.7–1.8 Å, in agreement with the dipole-driven inversion mechanism discussed in the literature.^2,9−11^ Another interesting observation is that for all inversion transition states the CO molecule is further away from the surface than the equilibrium C- and O-down configurations.

### 4.4 Modeling High CO Coverage

Experiments probing CO isomerization^2^ were performed under very high CO coverage where the CO molecules form a monolayer or a monolayer covered by multiple overlayers. In the following, we will examine the main differences in the potential energy surface at high CO coverage using model **5** and treating the interaction of the central CO molecule with the surrounding COs using the CO–CO potential of Chen et al.^51^ To account for the most important CO–CO interactions in a monolayer, pairwise interaction energies with the closest 12 neighboring COs (C-down) are added to the energy of model **5**. While limitations of the CO–CO potential do now allow us to study the convergence of this interaction, we find that going from 4 to 8 surrounding COs has an effect on the C-down and O-down interaction energies of 8.4 and 22.4 cm^–1^, respectively, while going from 8 to 12 COs changes the interaction energy by 2.0 and 3.2 cm^–1^, respectively.

To mimic the experimental conditions during vibrational energy pooling, we keep the geometry of the additional COs fixed at the equilibrium *R* distance (3.43 Å) and equilibrium bond length (*r* = 1.132 Å), while the central CO is allowed to stretch. This model does not correspond to the most stable arrangement of COs in a monolayer at low temperature (< 35 K); however, we expect that it still captures the essential features of the CO potential surface in a monolayer (especially considering the small energy difference between the tilted and perpendicularly aligned structures). Extending the current embedding study to the tilted case (even with two COs per unit cells^51^) is desirable; however, it would require the optimization of the tilted geometry, which in the absence of analytic energy gradients is significantly more expensive.

As shown in Figures 8A, B, there are several noticeable differences between the isolated and monolayer PESs: (1) The energy difference between the C-down and O-down configurations is predicted to increase. At *r* = 1.432 Å, Δ*E*_iso_ is −6.8 cm^–1^ for the isolated CO model and −309 cm^–1^ for the monolayer. This increase indicates that the presence of other CO molecules energetically favors the interchange between the C-down to O-down geometries, and the vibrational excitation level required for this transition to occur is likely to be lower than for an isolated CO. (2) The barrier for going from the C-down to O-down configurations is predicted to be slightly lower than in the case of isolated CO, especially at stretched *r*. For example, the inversion barrier for a monolayer at *r* = 1.432 Å is predicted to be ca. 523 cm^–1^, while for the isolated CO model it is only 630 cm^–1^.

In Table 4, we report computed adsorption energies (without zero-point vibrational corrections) using a 1-D scan along *R* for different *r* values. For CO at the equilibrium distance (*r* = 1.132 Å), the C-down adsorption energy predicted by model **5** is 1561 cm^–1^ (isolated CO) and 1722 cm^–1^ (1/1 coverage). The adsorption energy estimated with both the single CO and the 1/1 models is very close to the experimental monolayer adsorption energy from Richardson et al.^68^ (1504 cm^–1^) and the theoretical estimate of Chen et al.^9^ (ca. 1502 cm^–1^) for tilted CO. Our adsorption energy for an isolated CO also compares favorably with the low-coverage CO adsorption energy (without zero-point energy corrections) reported by Boese and Saalfrank (17.8 kJ/mol = 1488 cm^–1^).^7^ This adsorption energy was computed using an embedded NaCl_5_^4–^ cluster and MP2 extrapolated to the complete basis set limit and includes CCSD(T) and long-range corrections.

**Table 4:** Computed CO Adsorption Energy (without Zero-Point Vibrational Corrections) for the C-Down and O-Down Configurations of Isolated CO and a 1/1 CO Monolayer on NaCl(100)^a^

configuration	coverage	*r* (Å)	*R* (Å)	adsorption energy (model 5) (cm^–1^)
C-down	single	1.132	3.381	1561.2
O-down	single	1.132	3.131	1071.2
C-down	single	1.432	3.653	1186.9
O-down	single	1.432	3.236	1193.7
C-down	single	1.732	3.918	889.8
O-down	single	1.732	2.917	1391.8
C-down	1/1	1.132	3.376	1722.4
O-down	1/1	1.132	3.147	1299.9
C-down	1/1	1.432	3.615	1358.0
O-down	1/1	1.432	3.300	1667.4
C-down	1/1	1.732	3.870	952.2
O-down	1/1	1.732	3.448	2074.3

a For a given coverage case and *r* value, the corresponding equilibrium *R* value is obtained using the same fitting procedure used for the PES. The adsorption energy is relative to the energy of CO + NaCl(100) (estimated by a computation with *R* = 200 Å). Note that for O-down single-coverage computation at *r* = 1.732 Å, the tilted geometry is used as the global minimum.

An interesting question to ask is, will the increased isomerization barrier in a monolayer cause a CO molecule to favor desorption instead of isomerization to the O-down configuration? Referring to Figure 8B, within the monolayer CO model, at *r* = 1.432 Å the transition barrier from C-down to O-down is 523 cm^–1^, and the barrier from O-down to C-down is 832 cm^–1^. These barriers are significantly lower than the desorption energy and decrease as *r* increases. Note that the inversion barrier found here is an upper-limit estimate of the actual barrier (since we do not relax the remaining coordinates). In summary, CO inversion is predicted to be energetically more favorable than desorption in both low and high coverage situations, even for highly vibrationally excited CO. However, this information is not sufficient to conclude that inversion is kinetically more favorable than desorption.

### 4.5 Vibrational Analysis

In this section, we perform vibrational analyses using the CO-NaCl(100) potentials discussed in Section 4.3 and the three vibrational model Hamiltonians defined in Section 2.2, considering potentials expanded around the perpendicular geometry minimum. For each Hamiltonian, we fit the eigenvalues corresponding to vibrational states with stretching mode quantum number ν_*r*_ up to 25 to a cubic polynomial (for the fits of the 2D Hamiltonians, we select only states that are not excited along the inversion or desorption coordinates). The resulting vibrational constants ω_e_, ω_e_*x*_e_, and ω_e_*y*_e_ are used to compare these vibrational models.

As can be seen in the Table 5, the gas-phase ^13^C^18^O vibrational constants ω_e_*x*_e_ from our theory (12.3 cm^–1^) are in relatively good agreement with the experimental values (12.1 cm^–1^);^2^ however, ω_e_ and ω_e_*y*_e_ (2046.2 cm^–1^, 6.73 × 10^–3^ cm^–1^) are smaller than the experimentally determined ones (2067.8 cm^–1^, 9.1 × 10^–3^ cm^–1^). These results show the same trend observed in Table 1, where ω_e_ and ω_e_*y*_e_ are underestimated when dynamical correlation is treated at second order.

**Table 5:** Experimental and Theoretical Vibrational Constants of ^13^C^18^O for the CO–NaCl(100) Monolayer Case^a^

model	Hamiltonian	ω_e_ (cm^–1^)	ω_e_*x*_e_ (cm^–1^)	ω_e_*y*_e_ (10^–3^ cm^–1^)
gas phase				
Exp^b^		2067.80	12.07	9.1
this work	1D	2046.15 ± 0.12	12.27 ± 0.011	6.73 ± 0.29
CCSD(T)^c^	1D	2067.00 ± 0.03	12.11 ± 0.003	11.39 ± 0.08
isolated CO on NaCl(100)				
C-down, model 5	1D (*R* = 3.43 Å)	2060.47 ± 0.20	12.13 ± 0.018	12.36 ± 0.46
O-down, model 5	1D (*R* = 3.23 Å)	2041.04 ± 0.18	12.29 ± 0.016	9.14 ± 0.40
O-down, model 5	1D (*R* = 3.43 Å)	2039.81 ± 0.20	12.25 ± 0.018	6.65 ± 0.46
C-down, model 5	2D(*r*,θ)	2059.45 ± 0.16	12.10 ± 0.014	11.44 ± 0.36
O-down, model 5	2D(*r*,θ)	2040.23 ± 0.18	12.31 ± 0.016	8.15 ± 0.42
C-down, model 5	2D(*r*,*R*)	2061.11 ± 0.15	12.24 ± 0.014	6.65 ± 0.34
O-down, model 5	2D(*r*,*R*)	2042.56 ± 0.13	12.30 ± 0.012	8.56 ± 0.30
CO monolayer (1/1) on NaCl(100)				
C-down, Exp^b^		2075.7 ± 0.7	12.21 ± 0.05	11.5 ± 1.1
C-down, model 5 + CO–CO int^c^	2D(*r*,*R*)	2058.85 ± 0.13	12.14 ± 0.012	6.77 ± 0.30
O-down, Exp^b^		2058.8 ± 0.9	12.18 ± 0.08	9.2 ± 1.8
O-down, model 5 + CO–CO int^c^	2D(*r*,*R*)	2036.31 ± 0.13	12.37 ± 0.012	7.42 ± 0.29

a The theoretical constants are computed using different model potential energy surfaces.

b From ref (2).

c Includes the CO–CO interaction energy from the complete-basis-set limit all-electron CCSD(T)-F12b potential reported in ref (51).

**Figure 9:**
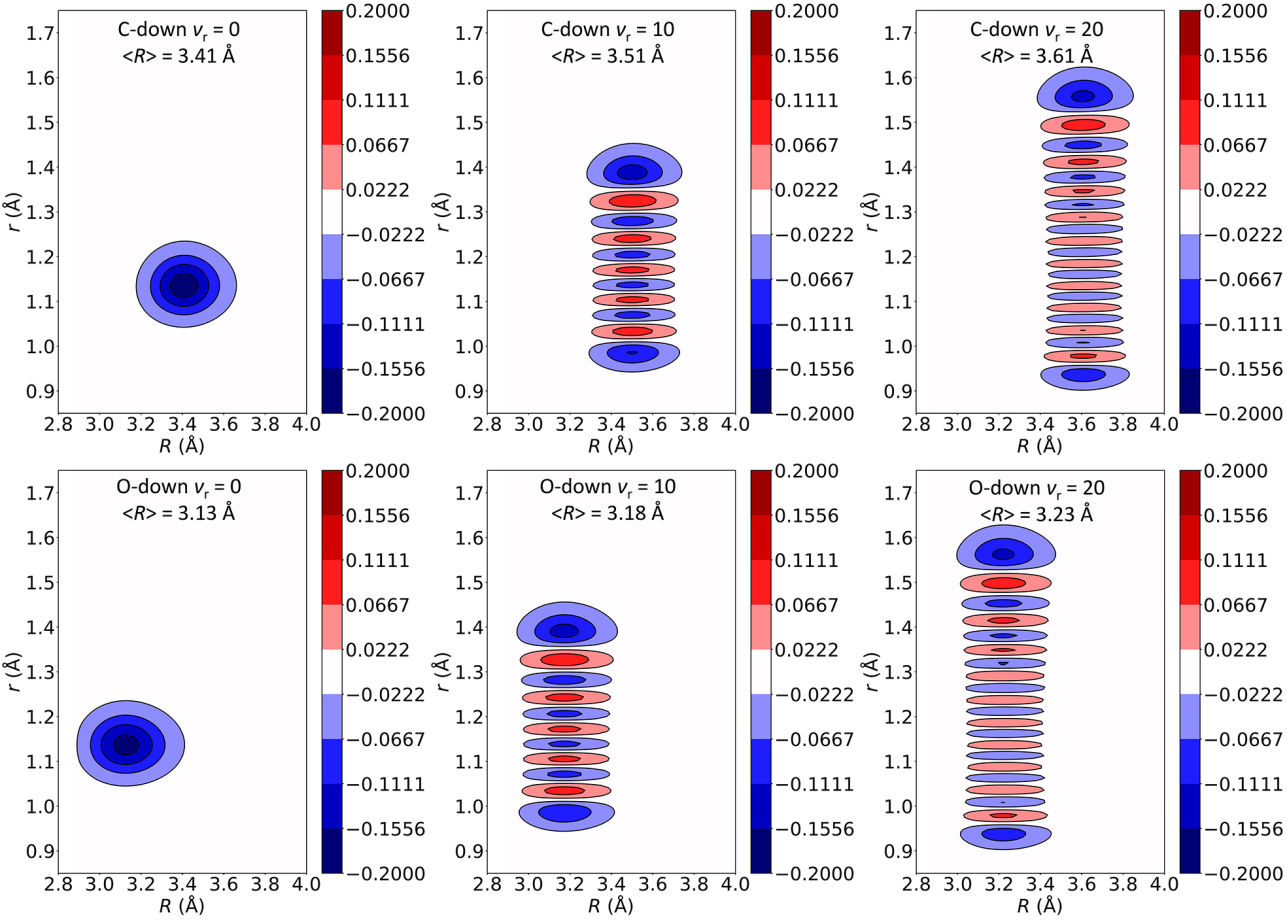
Vibrational wave functions for the ν_*r*_ = 0, 10, and 20 states of a isolated CO on a NaCl(100) surface. The wave functions are calculated using a two-dimensional model involving the CO stretching (*r*) and desorption (*R*) coordinates.

Next, we consider the vibrational spectrum of an isolated CO molecule computed with the 1D and 2D Hamiltonians. The 1D model for the C-down (at *R* = 3.43 Å with θ = 0°) and O-down (at *R* = 3.23 Å with θ = 180°) geometries already captures the qualitative red- and blue-shift of the CO stretching frequency for the C- and O-down geometries, observed experimentally in the monolayer^2^ and noted in previous theoretical studies.^9,10^ The 2D models allow us to study the effect of the inversion and desorption degrees of freedom on the CO vibrational states. As can be seen in Table 5, the vibrational constants obtained from the 2D(*r*,θ) and model are nearly identical with those from the 1D model, while those for the 2D(*r*,*R*) model show some more pronounced differences. For example, the ω_e_*y*_e_ constant at the C-down geometry goes from 12.36 × 10^–3^ cm^–1^ (1D model) to 11.44 × 10^–3^ cm^–1^ for the 2D(*r*,θ) model, while it decreases to only 6.65 × 10^–3^ cm^–1^ in the 2D(*r*,*R*) model. This observation indicates that the coupling between the CO stretching and translational motion is stronger than the one between the CO stretching and inversion modes, suggesting that the 2D(*r*,*R*) Hamiltonian should better capture the vibrational features of the CO–NaCl(100) system. This difference in coupling can be also observed from the corresponding 2D wave functions. As shown in Figure S3, in the 2D(*r*,θ) model the wave function remains localized around θ = 0 and 180° as ν_*r*_ increases from 0 to 20. Instead, as shown in Figure 9, the wave function for the 2D(*r*,*R*) model shifts to higher *R* values when the stretching mode is excited.

Lastly, we use the 2D(*r*,*R*) Hamiltonian to perform a vibrational analysis for the monolayer with 1/1 coverage using the potential from model **5** augmented with CO–CO pairwise interactions. The vibrational constants obtained from our vibrational analysis and experiment are shown in Table 5. Experiments show that going from gas-phase CO to the 1/1 coverage monolayer, the ω_e_ constant is blue-shifted by +7.9 cm^–1^ for the C-down isomer and red-shifted by −9 cm^–1^ for the O-down isomer.^2^ As shown in Table 5, the predicted ω_e_ values based on the additive CCSD(T) potential are 2059 and 2047 cm^–1^ for the C- and O-down CO, which correspond to ω_e_ shifts of +12.7 and −9.8 cm^–1^, in good agreement with the experimental shifts of +7.9 and −9.0 cm^–1^.^2^ Under this potential, the O-down CO state with ν_*r*_ = 16 has a lower energy (electronic plus vibrational) than the equivalent C-down CO with the same ν_*r*_. This value is smaller to the one previously reported by Nandi et al.^11^ (ν_*r*_ = 22) from quasi-classical trajectories using a 13-CO cluster model. The turning point of CO monolayer model for the state ν_*r*_ = 16 is around 1.32 Å, and the corresponding C-down to O-down barrier is ca. 680 cm^–1^. Even though the additive CCSD(T) CO–CO potential may be an oversimplified model of a CO monolayer on NaCl(100) due to the lack of surface polarization effects, the need for such higher-level treatment suggests the importance of this energetic contribution to simulations of the CO flipping mechanism. More importantly, we find it crucial to include the CO–CO interaction to stabilize the O-down isomer, in the absence of which we do not observe preference for the O-down configuration under ν_*r*_ = 25. This finding suggests then that the CO–CO interaction in the monolayer is an important enabler of the inversion mechanism observed in experiments.

## 5 Conclusion

In summary, we built a multireference quantum embedding model that allows us to simulate the highly vibrationally excited states of CO on a NaCl(100) surface. Our computational model for an isolated CO on NaCl(100) surface treats the fragment CO–NaCl_4_ at the level of second-order DSRG multireference perturbation theory, the Na_8_Cl_5_ environment at the mean-field level via ASET, and a surrounding Na_1755_Cl_1755_ layer as a classical electrostatic potential plus dispersion corrections. For an isolated CO molecule on a NaCl(100) surface, we find that the optimal geometry for both C-down and O-down isomers is perpendicular to the surface plane. We computed a three-dimensional potential energy surface involving the CO stretching mode, inversion mode, and center-of-mass translation mode. From the isolated CO–NaCl(100) PES, we confirm that the C-down configuration is preferable at smaller C–O bond distances. In contrast, for stretched C–O bond distances (> 1.6 Å), the O-down configuration becomes more stable. The PES shows significant variations with the center-of-mass distance. A vibrational analysis based on the isolated CO PES successfully reproduces the blue and red-shift of the CO stretching frequency at the C-down and O-down geometries, in agreement with previous experimental and theoretical studies.^2,9,10^

We also studied the CO–NaCl(100) system at high CO coverage with a model that combines our isolated CO potential and a highly accurate CO–CO potential.^51^ A vibrational analysis based on our high coverage potential predicts shifts in the CO stretching constants that are in near quantitative agreement of theory and experiment. Compared to the isolated CO on the surface, the CO–CO interactions in the monolayer decrease the energy difference between the O-down and C-down geometries and the isomerization barrier. Our results suggest that CO–CO interactions are not only necessary for accurate simulations of the CO inversion mechanism under experimental conditions (high coverage) but also play a crucial role in enabling this process by reducing the energy difference and barrier.

Our results show that the ASET embedding approach offers a simple and accurate way to extend multireference computations to molecules interacting with surfaces of insulators. This approach can treat bond dissociation processes and may be easily extended to electronically excited states. Nevertheless, several improvements could be made to this study, including on the electronic structure theory side, where the use of a larger basis and higher-order correlated methods would be desirable. An interesting future direction would be to extend the present analysis using the 3D model Hamiltonian from Sinha and Saalfrank to include the full coupling of the *r*, *R*, and θ degrees of freedom.^10^ However, because the energy minimum of the O-down configuration is both translated and rotated along the azimuthal axis, it may be necessary to go beyond even a three-dimensional model to capture the effect of these two additional degrees of freedom. In the future, we expect that the development of analytical gradients^69,70^ and fast higher-order correlation methods will help improve the accuracy and number of degrees of freedom that can be treated in simulations of molecule–surface interactions.

## Supporting Information Available

Details of the classical external corrections, Cartesian geometries of the QM cell and classical potential cells, the potential energy surface computed with multiple models, and vibrational wave functions (PDF)
The Cartesian coordinates of the atoms in the QM cell and the python script to generate classical potential (TXT)
Potential energy surface computed for the single-CO model 5 for ^12^C^16^O (XLSX)
Potential energy surface computed for the single-CO model 5 for ^13^C^18^O (XLSX)
Potential energy surface computed using the monolayer model (with 12 environment COs) for ^12^C^16^O (XLSX)
The jupyter-notebook for analyzing, fitting, and plotting the potential energy surfaces (ZIP)
