# Supplementary material for: CO Inversion on a NaCl(100) Surface: A Multireference Quantum Embedding Study
Source: J Phys Chem A. 2023 Feb 17;127(8):1975–87. doi: 10.1021/acs.jpca.2c05844 (PMC9986868; doi:10.1021/acs.jpca.2c05844)
Supplement: Supplementary file 1 — jp2c05844_si_001.pdf [file jp2c05844_si_001.pdf]

Supplementary Information: CO Inversion on a NaCl(100) Surface: A Multireference Quantum Embedding Study

Nan He, Meng Huang, and Francesco A. Evangelista*

Department of Chemistry and Cherry Emerson Center for Scientific Computation, Emory University, Atlanta, GA 30322, USA

E-mail: francesco.evangelista@emory.edu

Quantum cluster benchmark and convergence.

This appendix discusses how we construct the quantum mechanical cluster and external classical corrections to the DSRG-MRPT2 embedded in ASET(mf) potential. The quantum mechanical (QM) cell used in our computations is the Na_9Cl_9 cluster. We tested other clusters and compared the results using DFT(B3LYP). In Table. S1 we compared the optimized R, adsorption energy, and vibrational frequencies computed using $CONa_9Cl_9$, $CONa_{18}Cl_{18}$, and $CONa_{25}Cl_{25}$ cluster. The difference from extending the cluster can be up to 0.03 Åfor R, 165 cm^{-1} for the adsorption energy, and 3.4 cm^{-1} for frequencies. The Na_9Cl_9 quantum cell do capture the essential characteristics; however, to achieve better accuracy, the contributions of extended cells beyond Na_9Cl_9 should also be considered. These contributions will be addressed using the classical potential described in the following section.

Table S1: Comparison of different QM clusters using DFT(B3LYP). The value of R is optimized using a one-dimensional scan where all other coordinates are fixed. A.E. stands for CO adsorption energy. The ν_0 (C-d) and ν_0 (O-d) are computed using a 1-D DVR along r using 100 points between 0.83–1.83 Å. The potential used in the DVR computation is obtained by cubic spline interpolation of 21 equidistant points.

Model	Cluster	R (C-d)	R (O-d)	A.E.	ν_0 (C-d)	ν_0 (O-d)	$\Delta\nu_0$
**q1**	$CONa_9Cl_9$ $(3 \times 3 \times 2)$	3.46	3.26	585	2192.6	2185.6	7.0
**q2**	$CONa_{18}Cl_{18}$ $(3 \times 3 \times 4)$	3.46	3.26	612	2195.0	2183.3	11.7
**q3**	$CONa_{25}Cl_{25}$ $(5 \times 5 \times 2)$	3.44	3.23	750	2196.0	2183.8	12.2

Classical external corrections

We construct our external potential using a point charge model placing layers of Na_9Cl_9 clusters around the QM cell. The arrangement of the classical potential layers is shown in Figure SS1 A. The electrostatic interaction is also augmented with corrections for dispersion corrections using a pairwise correction. Note that the QM cell is not a classical unit cell; therefore, we surround it with two types of cells containing the atoms arranges in the same way but with opposite charges [MM(+) and MM(−)]. These classical point charges are

expanded layer-by-layer as shown in Figure SS1; each new layer extends both in (100) plane (XY) and in the (−1, 0, 0) direction (−Z). We use the charges +0.534 for Na^+ and −0.534 for Cl^-, as optimized in a previous work.[1] For the monolayer model, 20 additional C-down CO will be placed on the Na^+ site at $R = 3.43$ in the first classical layer. The charges used for C and O are -0.09886 and 0.09886, respectively.

The dispersion interactions contribution from atoms in the MM cell are also important in this system, especially from the atoms within the first MM layer. Since all short-range dispersion interactions are already handled in the embedding fragment using DSRG-MRPT2, the only energy correction term we need is the long-range attractive dispersion potential. Following Ref. 2, we approximate the dispersion interaction using the following equation:

$$U_{\text{disp}} = -\sum_{i \in \text{CO}} \sum_{j \in \text{env}} \frac{C_{ij}}{r_{ij}^6}, \tag{1}$$

where the C_6 dispersion coefficients C_{ij} are equal to 383.3, 3935.9, 256.6, and 2633.0 kJ/mol·Å^6 for $C\cdots Na^+$, $C\cdots Cl^-$, $O\cdots Na^+$, and $O\cdots Cl^-$, respectively. For model **5**, the summation over j includes atoms in the ASET(mf) environment (8 Na^+ and 5 Cl^- ions) plus all added classical layers. This formula is similar to the D2 correction commonly employed to correct approximate DFT functionals.[3]

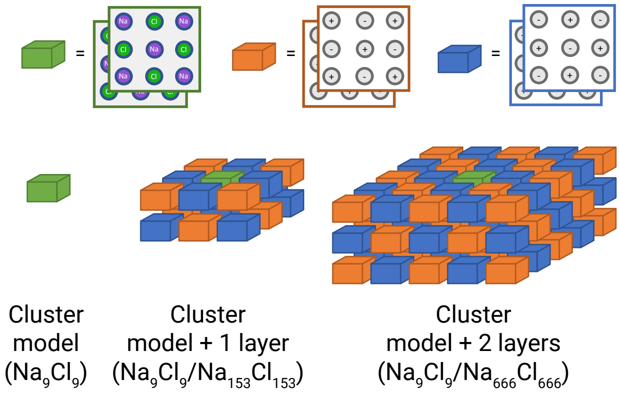

Figure S1: An illustration of the quantum-mechanical and classical potential regions of the multilayer model used in this work.

In Table S2, the adsorption energies and C-down frequencies computed using 0 4 layers

of classical potentials are shown.

Table S2: Test of the convergence of various properties vs. the number of classical potential layers. The distance of the C atom from the surface [R(C-d)] is optimized using a one-dimensional scan where all other coordinates are fixed. The adsorption energy excludes zero-point vibrational corrections. The fundamental CO stretching frequency for the C-down isomer [ν_0 (C-d)] is computed using a 1-D DVR along r using 100 points between 0.83–1.83 Å. The potential used in the DVR computation is obtained by cubic spline interpolation of 21 equidistant points.

Model	Classical potential	R (C-d) (Å)	Adsorption energy (cm^{-1})	ν_0 (C-d) (cm^{-1})
**L0** (model **2**)	Na_0Cl_0	3.41	1320	2130.8
**L1**	$Na_{153}Cl_{153}$	3.38	1566	2135.4
**L2**	$Na_{666}Cl_{666}$	3.38	1575	2135.7
**L3** (model **5**)	$Na_{1755}Cl_{1755}$	3.38	1576	2135.9
**L4**	$Na_{3636}Cl_{3636}$	3.38	1577	2135.8

Table S2 shows that the CO distance from the surface, the adsorption energy, and fundamental CO stretching frequency converge rapidly as layers of classical potential are added. In the computations described in the main text, we used three layers of classical charges to construct the potential (**L2** = model **5**) to guarantee that the adsorption energy and the fundamental frequency are converged to within 1 cm^{-1}.

Convergence of 2D DVR grid

To validate the convergence of our 2D DVR grid, we construct and diagonalize the 2D(r, R) vibrational Hamiltonian of $C^{13}O^{18}$ using a 2D grid with the same number of points (N_g) along both dimensions (for a total of N_g^2 basis functions). The absolute difference in the eigenvalue of the $\nu_r = 25$ state using N_g and $N_g - 1$ grid points ($N_g = 47 - 64$, with an increment of 1) are evaluated and plotted in S2. As it can be seen from this plot, energy differences after $N_g = 57$ are all smaller than 0.1 cm^{-1}.

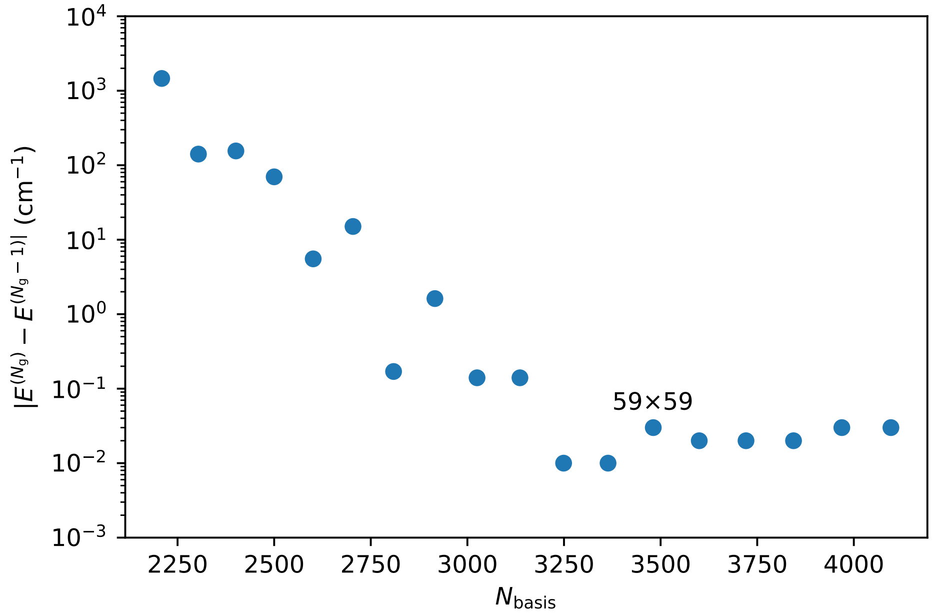

Figure S2: Convergence of the energy of the $\nu_r = 25$ vibrational state of CO as a function of the number of DVR grid points for the the 2D(r, R) vibrational Hamiltonian of $C^{13}O^{18}$. The plot shows the absolute difference in the eigenvalues computed with a 2D DVR grid consisting of N_g and $N_g - 1$ grid points (along both coordinates). 59×59 is the default number of grid points used in this study.

Cartesian coordinates

In the file `cells.txt`, we include

- The Cartesian coordinates of the atoms in the QM cell, including the partitioning of atoms between the fragment and environment.
- The coordinates and charges of the atoms that contribute to the classical potential. A python script to generate the classical charges are attached.

Potential energy surfaces for an isolated CO molecule and the monolayer (1/1 coverage) model of CO-NaCl(100)

We included:

- 3-dimension potential energy surfaces (PES) computed for the single-CO model **5** at the ASET(mf)-[DSRG-MRPT2]/cc-pCVTZ level of theory, for both $^{12}C^{16}O$ (`PES_singleCO_C12O16.xlsx`) and $^{13}C^{18}O$ (`PES_singleCO_C13O18.xlsx`). These two sets of data differ in the position of the center of mass.

- 3-dimensional PES computed using the monolayer model (with 12 environment COs) at the ASET(mf)-[DSRG-MRPT2]/cc-pCVTZ level of theory (`PES_monolayerCO_C12O16.xlsx`)

- All PESs include electrostatic potential and dispersion corrections generated by three layer of atoms (see main text for the definition). Both R and r are given in angstrom while the tilt angle is given in degrees.

- Note that for the monolayer PES, we report data only for the $^{12}C^{16}O$ isotopologue, since the CO is fixed at a perpendicular geometry and the center of mass is aligned with the central Na atom. The corresponding PES for $^{13}C^{18}O$ may be generated from the given values of r and R by a change of coordinate.

- The notebook for analyzing, fitting, and plotting the PESs are also attached as PES_Analysis.ipynb in PES_Analysis.zip. It includes six sections encompassing all computations we have done on those PESs. The notebook is well commented. The input for the notebook consists of the .xls files of the PESs. They are also attached in the .zip folder.

Vibrational wave functions calculated using the 2D(r, θ) Hamiltonian model for isolated CO on NaCl(100) surface

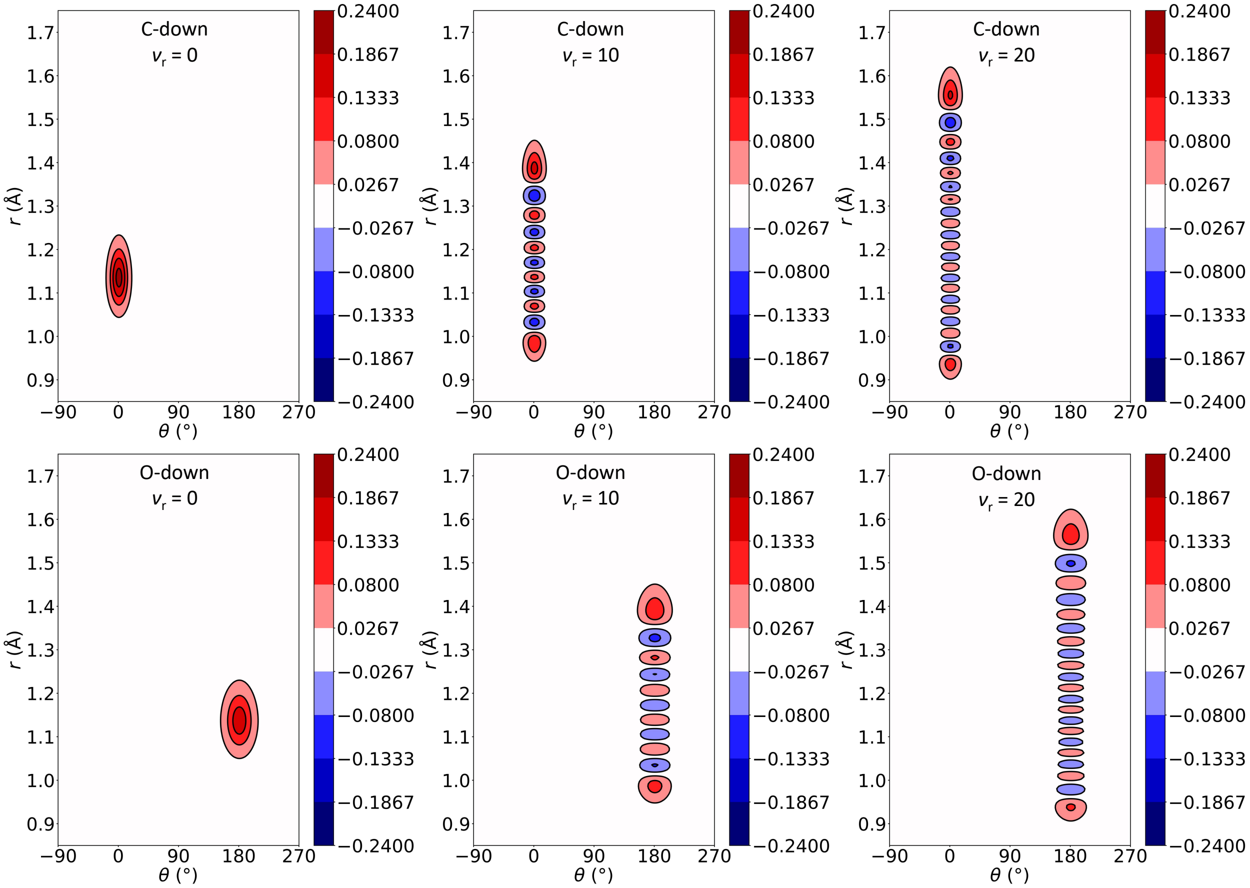

Figure S3: Vibrational wave functions for the $\nu_r = 0$, 10 and 20 states of an isolated CO on a NaCl(100) surface. The wave functions are calculated using a two-dimensional model involving the CO stretching and inversion coordinates.

References

(1) He, N.; Evangelista, F. A. A zeroth-order active-space frozen-orbital embedding scheme for multireference calculations. *J. Chem. Phys.* **2020**, *152*, 094107.

(2) Meredith, A. W.; Stone, A. J. A perturbation theory study of adlayer CO on NaCl(100). *J. Chem. Phys.* **1996**, *104*, 3058–3070.

(3) Grimme, S. Semiempirical GGA-type density functional constructed with a long-range dispersion correction. *J. Comput. Chem.* **2006**, *27*, 1787–1799.
